# Inappropriate Rehabilitation Claims and Financial Burden in Japan: Analysis of Reports from the Board of Audit of Japan

**DOI:** 10.31662/jmaj.2025-0313

**Published:** 2026-04-03

**Authors:** Daisuke Kubo, Takuya Hirose, Yushi Asaoka

**Affiliations:** 1 Department of Health Data Science, Yokohama City University, Kanagawa, Japan; 2 Department of Rehabilitation, Shonan-Keiiku Hospital, Kanagawa, Japan; 3 Graduate School of Health Science, Kibi International University, Okayama, Japan; 4 Physical Therapy Course, Department of Rehabilitation, Faculty of Health Science, Nihon Institute of Medical Science, Saitama, Japan

**Keywords:** rehabilitation, health expenditures, fraud, medical audit, Japan, inappropriate claims

## Introduction

Healthcare fraud is a critical issue that imposes a severe economic burden on the integrity of healthcare systems. One global analysis based on measured loss rates across multiple countries estimated that approximately US$415 billion (€301 billion; £259 billion) in health spending is lost to fraud (and error) annually ^(1)^. Such inappropriate billing practices lead to the inefficient utilization of healthcare resources. This incurs social losses by funding treatments that do not improve patient health ^(2)^.

Although healthcare fraud is a global challenge, its full scope remains difficult to grasp, and related research reports are limited ^(3), (4)^. Previous studies on inappropriate billing for hospital-acquired infections have reported that over 10,000 (18.5%) of approximately 60,000 annually billed present-on-admission infections were actually upcoded hospital-acquired infections, leading to an additional cost burden of 200 million USD for Medicare ^(5)^. In addition, healthcare fraud has been documented across emergency and critical care, surgery, and anesthesia ^(6)^.

Inappropriate claims for rehabilitation services have also been reported. Indeed, prior studies have shown that fraudulent practices, such as shortening therapy sessions while charging for the full duration and billing individual rates for group therapy, are not uncommon, with 81% of surveyed therapists admitting to at least one type of Medicare/Medicaid fraud ^(7)^. However, prior research on fraud in this field has primarily examined the nature of such practices, meaning the financial scale and detailed patterns have not been fully elucidated.

In rapidly aging Japan, national medical expenditures have exceeded 45 trillion Japanese yen (JPY) annually since fiscal year 2021. Rehabilitation medicine, which is essential for maintaining patient function and quality of life, is growing in importance, and the appropriate management of its costs has become an urgent issue. In Japan, rehabilitation fees are calculated based on detailed criteria that vary according to the patient’s condition and treatment duration, necessitating frequent documentation and adjustments. This complexity, combined with the high demand for services in the aging population, may create opportunities for inappropriate claims if oversight is insufficient. Although fraudulent practices in rehabilitation services have been documented in the United States, little is known about the occurrence of fraud in rehabilitation billing under Japan’s public health insurance system. As such, addressing this gap is crucial for ensuring the financial soundness of the insurance system and protecting patient interests. This study aimed to clarify the real-world status of inappropriate billing for rehabilitation fees in Japan.

## Materials and Methods

### Study design

This descriptive investigation aimed to clarify the status of inappropriate billing for rehabilitation fees under Japan’s public health insurance system.

### Data sources and collection

This study targeted audit reports from the Board of Audit of Japan from fiscal years 2019 to 2023, which identified inappropriate government burdens related to medical expenses. The Board of Audit of Japan operates as a constitutional organization independent of the Cabinet, with the primary mission of auditing the final accounts of state expenditures and revenues and submitting audit reports to the Diet. We obtained audit reports from the Board of Audit of Japan’s Audit Report Database ^(8)^ using the Japanese keyword “リハビリテーション料” (translation: rehabilitation fee). Reports containing explicit descriptions of inappropriate billing for rehabilitation services were also included. Reports from fiscal years outside 2019-2023 or addressing topics unrelated to medical expenses were excluded. The 2019-2023 period was chosen because the medical service fee schedule is revised every two years, while including older data could lead to discrepancies with the current reimbursement framework. The data collected for each fiscal year included the total number of audited medical institutions. This figure includes all facilities examined, regardless of whether inappropriate rehabilitation claims were identified. Additional items included the number and amount of excessive medical expenses and the inappropriate government burden. We further collected details on excessively paid circumstances in this study. The details of excessive payments are described in the audit reports as a summary of the major fraudulent billing situations revealed in the audits for each fiscal year. These details reflect the overarching patterns described in the reports, but do not represent a comprehensive classification of all inappropriate billing cases. The categorized examples presented in this study are derived from these descriptive summaries and are intended to illustrate the main types observed.

### Analysis

For the collected data, the number of medical institutions, the number of excessively paid medical expenses, the amount of excessively paid medical expenses, and the amount of inappropriate government burden were described for each fiscal year. All monetary amounts are reported in JPY. Textual data from the details of excessively paid circumstances were categorized based on their similarity, and the frequency of each category was counted. Two researchers (DK and TH) independently reviewed the textual descriptions in the audit reports and inductively derived the initial categories from the observed claim patterns. Definitions were developed for each category to ensure clarity and consistency. To enhance the validity of the categorization, these categories were mapped onto a broader fraud-classification framework previously reported in the literature ^(9)^. Any discrepancies in this classification were resolved through discussion, and when a consensus could not be reached, a third researcher (YA) was consulted to make the final decision. Although inter-rater reliability statistics were not calculated in this study, the validity was supported by deductively mapping emergent categories to established typologies in prior research. Microsoft^Ⓡ^ Excel for Mac (version 16.98; Microsoft, Redmond, WA, USA) was used for data tabulation and analysis.

## Results

This study analyzed five audit reports. For FY2023, the values for the number of audited medical institutions, the number of excessively paid medical expenses, the amount of excessive medical expenses, and the amount of inappropriate government burden are presented as reference values, as they include fee categories beyond rehabilitation claims.

### Overview of inappropriate billing cases and amounts

Table 1 presents an overview of inappropriate billing cases and amounts for the period from fiscal years 2019 to 2023. A total of 99 medical institutions were audited, in a process not limited to rehabilitation services. The total number of excessively paid medical expenses amounted to 37,377 cases, totaling 379,128,361 JPY. Of this amount, the inappropriate government burden was 151,190,974 JPY. On average, each institution (n=99) incurred approximately 378 cases of excessive medical expenses. When restricted to rehabilitation, the average costs of excessively paid medical expenses and inappropriate government burdens amounted to 3.83 million JPY and 1.53 million JPY, respectively. Overall, the rehabilitation-related inappropriate government burden accounted for 39.9% of all excessively paid rehabilitation expenses.

**Table 1.** Number of Audited Medical Institutions, Cases, and Financial Results.

Item	FY2019	FY2020	FY2021	FY2022	FY2023	Total
Number of Audited Medical Institutions, n	15	39	24	21	28	99 (127)
Number of Excessively Paid Medical Expenses, n	8,261	16,800	7,290	5,026	(16,225)	37,377 (53,602)
Annual Excessively Paid Medical Expense totals, JPY	49,195,724	128,395,528	80,371,172	121,165,937	(153,456,801)	379,128,361 (532,585,162)
Annual Inappropriate Government Burden amounts, JPY	20,532,242	52,140,111	30,696,523	47,822,098	(58,051,425)	151,190,974 (209,242,399)

Note: Values in parentheses are listed as reference values as they include claims/reimbursements for medical management, first/revisit fees, hospitalization, meal/living care, treatment, and home medical care, in addition to rehabilitation. FY: fiscal year; JPY: Japanese yen.

### Frequency of detailed circumstances of inappropriate billing

Textual data from the details of excessively paid circumstances were categorized based on their similarity. The frequency represents the number of times each billing pattern appears in the audit reports. The most frequently reported category was non-reduction in patients with certified long-term care insurance, exceeding the standard calculation limits, with five cases identified. This was followed by billing for rehabilitation fees owing to the false onset of new diseases and billing exceeding the standard calculation days for patients without expected improvement from continued treatment, each with two cases. Billing for ineligible patients was reported in one case. Table 2 presents a breakdown and frequencies of these categories.

**Table 2.** Summary of Improper Billing Categories and Audit Findings.

Code	Category	Category Definition	Example Quote	Frequency (n)
“Improper coding and upcoding”	Non-reduction for patients under long-term care insurance exceeding standard claims periods	Claiming reimbursement for a service or procedure at a higher level than that provided.	“For eligible patients who are insured under long-term care insurance and received rehabilitation exceeding the standard number of days, rehabilitation fees were billed at the standard rate, instead of at the lower rate required.”	5
“Falsifying documents”	Billing for rehabilitation fees based on the false onset of new diseases	Certificates were falsified to demonstrate the medical necessity of a particular procedure to justify payment.	“For patients who experienced a single onset of a disease or condition, rehabilitation fees were repeatedly billed―even after the standard number of days―by falsely documenting a new onset of a disease or condition in the claims’ remarks column. This allowed for the continuous billing of a rehabilitation fee that is otherwise limited to the standard number of days.”	2
“Providing unnecessary care and maximizing care.”	Billing exceeding standard calculation days for patients unlikely to improve with continued treatment	Provision of medically unnecessary services, or the delivery of care in a greater volume than required for a patient’s treatment.	“Rehabilitation fees were billed for a duration exceeding the standard number of days, even when the patients did not meet the established eligibility criteria, such as those for whom continued treatment was expected to result in an improvement of their condition.”	2
	Billing for non-eligible patients	Providing services to ineligible patients.	“Rehabilitation fees were billed for patients who did not meet the eligibility criteria.”	1

Note: Data aggregated from fiscal year 2019 to 2023. The Code column presents the typologies of fraudulent billing established in previous research ^(9)^, while the Category column shows the specific billing categories identified in the audit reports. Frequencies indicate the number of times each billing pattern was described in the audit reports. The examples in this table are illustrative and based on descriptive summaries provided in the audit reports. They do not represent a full classification of all excessive billing cases.

## Discussion

This study investigated specific cases of inappropriate billing in Japan. The results indicate that inappropriate billing for rehabilitation fees was reported in all fiscal years. The most common details of excessively paid circumstances were non-reduction for patients with certified long-term care insurance, exceeding standard calculation limits.

The types of inappropriate billing for rehabilitation fees identified in this study, with non-reduction in patients with certified long-term care insurance exceeding standard calculation limits being the most frequent, along with billing for rehabilitation fees owing to the false onset of new diseases, billing exceeding standard calculation days for patients without expected improvement from continued treatment, and billing for ineligible patients, suggest that the complexity of medical fee billing is a contributing factor. “Upcoding,” wherein medical institutions inaccurately report diagnostic codes for higher reimbursements, has also been reported in other medical fields ^(5), (6), (9)^. In the field of rehabilitation, the diverse criteria for calculating medical fees and the need for flexible responses based on patient conditions are thought to increase the risk of inappropriate billing. A lack of education and training in billing practices among healthcare professionals, as well as the increasing complexity of billing systems and administrative burdens, are also suggested as contributing factors to inappropriate billing ^(10)^.

This study had several limitations. Firstly, the analysis was only descriptive, and inferential statistics were not used. Furthermore, our analyses were based on audit report data from FY2019 to FY2023 and reflected only the institutions that were selected for the audit. Therefore, the findings should be interpreted within this setting and period, and caution should be exercised when generalizing the results to other years or unaudited providers. In addition, the Board of Audits’ procedures for selecting audit targets and documenting findings are not publicly disclosed. This lack of transparency precludes the assessment of potential selection or reporting bias in the audit data.

This study examined inappropriate billing for rehabilitation under Japan’s public insurance system using audit reports. Across the 99 audited medical institutions, there were 37,377 cases of excessive medical expenses in rehabilitation, totaling 379,128,361 JPY, of which 151,190,974 JPY represented an inappropriate government burden. The most frequently described inappropriate claims pattern was non-reduction for patients with certified long-term care insurance for whom services extended beyond the standard duration typically covered. Medical institutions should thoroughly verify claim eligibility and adhere to the standard calculation periods before submission. Claims review agencies should conduct rigorous reviews of submitted claims to ensure compliance and identify any potential inconsistencies in rehabilitation billing.

## Article Information

### Acknowledgments

The authors express their gratitude toward the Board of Audit of Japan for providing access to their Audit Report Database, which was essential for this study. We would like to thank Editage (www.editage.jp) for English language editing.

### Author Contributions

Made substantial contributions to the conception and design of the study and to the acquisition, analysis, and interpretation of data: Daisuke Kubo. Contributed substantially to the conception, design, analysis, and interpretation of this study: Takuya Hirose. Contributed substantially to the conception, design, analysis, and interpretation of this study: Yushi Asaoka. All authors contributed to drafting the work or critically reviewing it for important intellectual content, provided final approval of the version to be published, and agreed to be accountable for all aspects of the work in ensuring that questions related to the accuracy or integrity of any part of the work were appropriately investigated and resolved, thereby meeting the four criteria for authorship defined by the International Committee of Medical Journal Editors (ICMJE).

### Conflicts of Interest

Daisuke Kubo declares a potential conflict of interest owing to his audit-support role in the public sector. All other authors declare no conflicts of interest.

### IRB Approval Code and Name of the Institution

This study was conducted using publicly available data; therefore, it was exempt from ethical review under the “Ethical Guidelines for Life Sciences and Medical Research Involving Human Subjects.”
